# MHC/class-II-positive cells inhibit corticosterone of adrenal gland cells in experimental arthritis: a role for IL-1β, IL-18, and the inflammasome

**DOI:** 10.1038/s41598-020-74309-0

**Published:** 2020-10-13

**Authors:** Hubert Stangl, Anita Krammetsvogl, Martin Lesiak, Christine Wolff, Rainer H. Straub

**Affiliations:** Laboratory of Experimental Rheumatology and Neuroendocrine Immunology, Department of Internal Medicine, University Hospital Regensburg, Biopark I, Am Biopark 9, 93053 Regensburg, Germany

**Keywords:** Rheumatic diseases, Rheumatology

## Abstract

In experimental arthritis, glucocorticoid secretion is inadequate relative to inflammation. We hypothesized that IL-1 is a key factor for inadequate glucocorticoid secretion in arthritic rats. Collagen type II—induced arthritis (CIA) in DA rats was the model to study effects of IL-1 on adrenal function. In the CIA model, an increase of intraadrenal MHCII-positive cells was observed. MHCII-positive cells or bone marrow-derived dendritic cells inhibited glucocorticoid secretion of adrenal gland cells. IL-1, but also IL-18 and the inflammasome were critical in glucocorticoid inhibition. Arthritic compared to control adrenal gland cells produced higher amounts of CXC chemokines from MHCII+ adrenal cells, particularly CINC-2, which is strongly dependent on presence of IL-1. In CIA, macrophages and/or dendritic cells inhibit glucocorticoid secretion via IL-1 in adrenal glands. These findings show that activated macrophages and/or dendritic cells inhibit glucocorticoid secretion in experimental arthritis and that IL-1β is a decisive factor.

## Introduction

In clinical rheumatology, endogenous glucocorticoid secretion is known to be too low to combat inflammation in patients with chronic inflammatory systemic diseases like rheumatoid arthritis (RA)^1,2^. However, endogenous glucocorticoid secretion would be important to contain the inflammatory process which has been demonstrated in RA^3^. Near normal but inadequate levels of glucocorticoids are present already early during rheumatic diseases^4–6^.

While injection of proinflammatory cytokines into healthy subjects produces a strong reaction of the hypothalamic-pituitary adrenal (HPA) axis with a huge cortisol surge, this response is missing after repeated injections over 3 weeks^7–9^. The same phenomena possibly support an inadequate HPA axis response in the presence of high inflammation in rheumatic diseases^1^. The reasons for these changes of the HPA axis on the level of the hypothalamus, pituitary and adrenal gland are not understood (this present study focuses on the adrenal gland).

The influence of cytokines on adrenal steroid hormone secretion has been detected early after discovery of proinflammatory cytokines (reviewed in Ref.^10^). For example, TNF exerts a direct inhibitory influence on expression of important steroidogenic enzymes in adrenocortical cells^11^. Anti-TNF therapy in RA can normalize some aspects of steroidogenesis^12^. The influence of cytokines on steroidogenesis has been reviewed elsewhere^13,14^.

In addition to cytokines, under normal and also under pathological conditions, macrophages infiltrate the adrenal cortex of rodents and humans^10^. Interestingly, macrophages and dendritic cells (DCs) are constitutively present in endocrine glands^15–19^, and they install an immediate augmentation response upon proinflammatory stimuli to interfere with these glands^18,20^. While these before mentioned responsiveness of endocrine glands is physiological in short-lived inflammation, the situation might largely change during a long-standing inflammatory process^21^.

In experimental arthritis in rats (unfortunately, there is no access to human material of adrenal glands), corticosterone and adrenocorticotropic hormone (ACTH) levels were only elevated on day 1–5 after immunization but were in the normal range from day 5 to 55 (Ref.^22^). Similar as in patients with RA, serum levels of corticosterone relative to proinflammatory cytokines were markedly lower in rats with arthritis than in controls (disproportion principle). In addition, number of altered swollen and cavitated mitochondria increased during the course of arthritis (maximum on day 55), which is an important sign for disruption of corticosteroid secretion^22^. IL-1 seems to stimulate some alterations^23,24^, but the role of intraadrenal macrophages or DCs has never been investigated in experimental arthritis^22^.

Based on these observations and on our earlier work^22^, we hypothesized that IL-1 is a key factor for inadequate glucocorticoid secretion in arthritic rats through inhibition of steroidogenesis, and that macrophages/DCs play an important role in inhibiting glucocorticoid secretion from adrenal gland cells.

## Patients, materials and methods

### Animals and induction of arthritis

Female Dark-Agouti rats (8–10 weeks of age) were purchased from Janvier (France). Five animals were housed in one cage, and they had ad-libitum access to standard chow and water. Animals were acclimated to the environment for at least 1 week before commencement of experiments. Arthritis was induced on day 0 by a single injection of 300 μg bovine type II collagen at the base of the tail (mdbioproducts, Egg bei Zurich, Switzerland) emulsified in an equal volume of incomplete Freund adjuvant (Sigma Aldrich, Deisenhofen, Germany) intradermally at the base of the tail as described previously^25^. Rats were inspected for clinical symptoms and weight on a daily basis. The Government of the Oberpfalz approved all experiments according to institutional and governmental regulations for animal use (Az. 54-2532.1-25/13, Az. 55.2-2532-2-413).

### Histological quantification of MHC class II: positive cells in adrenal glands

Rats from various time points during experimental arthritis and respective age matched control animals were killed by CO_2_ asphyxia. Adrenal glands were collected and subsequently fixed in phosphate buffered saline (PBS) solution containing 3.7% formaline on ice. Organs were dehydrated in PBS containing 20% sucrose and frozen in Tissue Tek (Sakura Finetek, The Netherlands) on liquid nitrogen. Frozen sections (8–10 µm thick) were obtained with a microtome (Leica, Germany), and after blocking sections were incubated with a primary antibody directed against MHC class II (MHCII) (ab23990, abcam, UK) and an Alexa Fluor fluorescent dye labeled secondary antibody (Invitrogen). Cell nuclei were counterstained with 4′,6-Diamidino-2-phenylindole (DAPI, Sigma Aldrich). With the help of a fluorescence microscope (Leica, Germany), the mean density of MHCII positive cells was determined in 17 randomly selected high power fields from at least three adrenal gland sections per animal.

In further histological studies of rat adrenal gland, we tried to detect B lymphocytes (anti-B220, Cat# 17-0460-82, Thermo Fisher Scientific, Dreieich, Germany), T lymphocytes (anti-CD3, Cat# 13-0030-82, Thermo Fisher Scientific), neutrophils (anti-neutrophil elastase, Cat# 21595, Abcam, UK) combined with a respective Alexa Fluor dye labeled secondary antibody (Invitrogen).

### Flow cytometric analysis of adrenal gland cells

For flow cytometric analysis, 5 × 10^5^ cells were stained with phycoerythrin (PE) Mouse anti-Rat MHC Class II RT1B RPE, (MCA46PE) and Fluorescein Isothiocyanate (FITC) Mouse anti-Rat CD86 (MCA2121F) for 30 min in the dark at 4 °C. Unspecific background staining was controlled by incubating the cells with the respective isotype controls RPE Mouse IgG1 (MCA1209PE) and FITC Mouse IgG1 (MCA1209F, all antibodies from Abd Serotec/Bio-Rad). Cell surface expression of MHCII and CD86 was analyzed using a FACS Calibur (Becton Dickinson Immunocytometry Systems, San Jose, California), and FACS data were analyzed with FlowJo software (Tree Star Inc, Ashland, Oregon).

### Isolation and culture of adrenal gland cells

Adrenal glands from control and arthritic rats were aseptically collected and subsequently submerged in ice-cold Hank’s balanced salt solution (HBSS) supplemented with 10% fetal bovine serum, 1% penicillin–streptomycin (P-S) solution (all from Sigma Aldrich) and 2 mg/ml Ciprofloxacin (Fresenius Kabi). Under sterile conditions, glands were freed from surrounding fat and connective tissue, briefly minced with a scalpel and then incubated for 1 h at 37 °C on a shaker in a dissociation solution containing 3 mg/ml Collagenase (C0130), 5 mg/ml BSA (A8806) and 0.1 mg/ml DNase (D5025-375KU, all from Sigma Aldrich).

The cell suspension was passed through a 70 µm mesh cell-strainer, and erythrocytes were lysed with a buffer (Qiagen, The Netherlands). Cell number and viability were checked by trypan blue exclusion. A total of 1.5 × 10^5^ cells per well were seeded into a 24 well plate (Corning) coated with Poly-D-lysine (Sigma Aldrich) in medium (DMEM-F12, D6434) supplemented with 1%P-S, 2 mg/ml, Ciprofloxacin (Fresenius Kabi), 2.5% fetal bovine serum and 15% horse serum (all from Sigma Aldrich) for subsequent cultures or co-cultures. Medium was changed on day 1, 3 and 5 of culture, and supernatants from day 3 and 5 were pooled and stored at − 20 °C for proteome analysis. This method was adapted and modified from various protocols^26–28^.

### Generation of bone marrow derived dendritic cells (BMDC)

Rats were killed by CO_2_ asphyxia. Femur and tibia bones from arthritic (CIA day28) and age matched controls were aseptically dissected. Under sterile conditions whole bone marrow was flushed out with RPMI-1640 Medium (R7509, Sigma Aldrich) supplemented with 2 mM glutamine, 1%penicillin/streptomycin solution, 10% fetal bovine serum, 10 mM HEPES, and 50 µM 2-mercaptoethanol (all from Sigma Aldrich), and collected after passing a 70 µm mesh cell strainer. After erythrocyte lysis (Qiagen), cell number and viability was checked by trypan blue exclusion. 1.5 × 10^6^ cells per well were seeded in 6-well plates (3516, Corning) in medium (see above) supplemented with 5 ng/ml recombinant GM-CSF, 5 ng/ml recombinant IL-4 and 25 ng/ml mFLT3-ligand (all from Peprotech, Rocky Hill, NJ, USA). Medium was changed on day 3 and 6 after seeding. On day 10, cells were carefully detached by pipetting, and passaged onto new plates. Medium was changed again on day 12, supplemented with 2.5 ng/ml GM-CSF only. On day 14 loose cell-clusters with dendritic shaped cells were collected by pipetting, and subsequently used for co-culture or in vivo experiments. The method was adapted and slightly modified from various protocols^29–32^.

### Co-culture of adrenal gland cells and BMDCs

Adrenal gland cells pooled from control rats (pooled from a total of n = 8–28 control animals depending on the size of the experiment) were seeded into 24 well plates as described, and on day 4 of culture, 0.75 × 10^5^ differentiated BMDCs from control or arthritic rats (not pooled) were added to the wells with co-culture inserts with 0.4 µm pore width (141002, Thermo Fisher scientific). After a co-culture period of 1 day, inserts including BMDCs were discarded, and adrenal gland cells were stimulated with 1 nM synthetic ACTH (Tetracosactid, Sigma-Tau) in DMEM-F12 supplemented with 2 mg/ml Ciprofloxacin (Fresenius Kabi), 1% P-S, 0.2% BSA and 1% glutamine (all from Sigma Aldrich) for 6 h. The level of corticosterone in supernatants was determined by ELISA (DEV9922, Demeditec, Germany).

### Co-culture of adrenal gland cells and intraadrenal MHCII+ cells

On day 0, adrenal gland cells pooled from control or arthritic (CIA day 28) rats (pooled from a total of n = 8–28 animals depending on the size of the experiment) were separated into MHCII− and MHCII+ fractions according to the manufacturer’s protocol (Anti-MHC Class II (OX6) MicroBeads, 130-090-759, Miltenyi Biotec, Germany). A total of 0.15 × 10^5^, 0.3 × 10^5^, 0.75 × 10^5^, or 1.5 × 10^5^ MHCII+ cells from control or arthritic rats were then seeded into inserts placed in wells from 24 well plates containing 1.5 × 10^5^ MHCII− or unseparated adrenal gland cells per well. Culture medium was exchanged on day 1, 2 and 4. Supernatants from day 2 and 4 were pooled per well and kept for proteome analysis. On day 4, inserts were removed, and remaining adrenal gland cells were stimulated with 1 nM ACTH for 6 h and levels of corticosterone in supernatants was determined by ELISA as described.

### Quantification of cytokines and chemokines in plasma and cell culture supernatants

Qualitative cytokine expression in supernatants from adrenal gland cells and BMDCs was analyzed with the proteome profiler rat cytokine array kit (ARY008, R&D systems). Levels of IL-1β, tumor necrosis factor (TNF), interleukin-10 (IL-10), interleukin-18 (IL-18), cytokine-induced neutrophil chemoattractant-1,-2,-3 (CINC-1,-2,-3), LPS induced chemokine (LIX), and tissue inhibitor of metalloproteinases 1 (TIMP-1) in cell culture supernatants and plasma samples of control and arthritic rats were determined by ELISA (IL-18 from Thermo Fisher; all others: DuoSets from R&D systems).

### Stimulation of adrenal gland cells with cytokines

Adrenal gland cells from control or arthritic animals were cultured as described and stimulated with either 1 ng/ml IL-1β (Peprotech), 100 ng/ml IL-1ra (Anakinra, Swedish Orphan Biovitrum), 0.01/1/10 ng/ml IL-18, or 10 µM NLR family pyrin domain containing 3 (NLRP3) inflammasome inhibitor MCC950 for three days. In experiments with IL-1β stimulation, supernatants from medium changes were kept for analysis of cytokine protein expression. In other experiments, cells were stimulated with 1/10/100 ng/ml CINC-2 or 0.1/1/25 ng/ml LIX (all from R&D systems, Wiesbaden, Germany) for 24 h. After a total culture period of 5 days in all experiments, cells were stimulated with 1 nM synthetic ACTH in medium and corticosterone secretion was determined as described.

### Stimulation of BMDCs with IL-1β and IL-1ra

BMDCs were generated as described, and 1 × 10^5^ BMDCs per well were then seeded into 24 well plates in medium without GM-CSF or IL-4, but supplemented with either IL-1β (1 ng/ml) alone, or in combination with IL-1ra (1, 10, 100 ng/ml). On day 2 and 5 after seeding, medium was exchanged, and supernatants were pooled and collected for proteome analysis.

### Neutrophil elastase staining

In order to test possible infiltration of adrenal glands with neutrophil granulocytes, frozen sections of adrenal glands from control and arthritic rats were stained with an anti-neutrophil elastase antibody (Abcam ab 21595) and a Alexa Fluor dye labeled secondary antibody (Invitrogen). Cell nuclei were counterstained with DAPI. The mean density of elastase positive cells was determined in 17 randomly selected high power fields from at least three adrenal gland sections per animal.

### PKH26 staining of BMDCs and i.v. application in vivo

BMDCs were generated from control and arthritic animals as described, and subsequently stained with the PKH26 red fluorescent cell linker kit for general cell membrane labeling according to the manufacturer’s protocol (PKH26GL, Sigma Aldrich). After staining, cells were washed twice with PBS. A total of 10 × 10^6^ cells per animal was injected into the tail vein. After 1 h, animals were killed, and lymph nodes, spleens and adrenal glands were fixed in PBS containing 3.7% formaline. The mean density of PKH26 positive cells was determined in 17 randomly selected high power fields from at least three sections per organ and animal.

### Statistical analysis

Data are given as line graphs or box plots with the 10th (whisker), 25th, 50th (median), 75th, and 90th (whisker) percentile. The medians of two groups were compared by Mann–Whitney U test (SigmaPlot). Medians of more than two groups were compared with ANOVA on ranks test (Sigma Plot). A p-value < 0.05 was the significance level.

## Results

### Appearance of MHCII+/CD86+ cells in adrenal glands during arthritis

**Figure 1:**
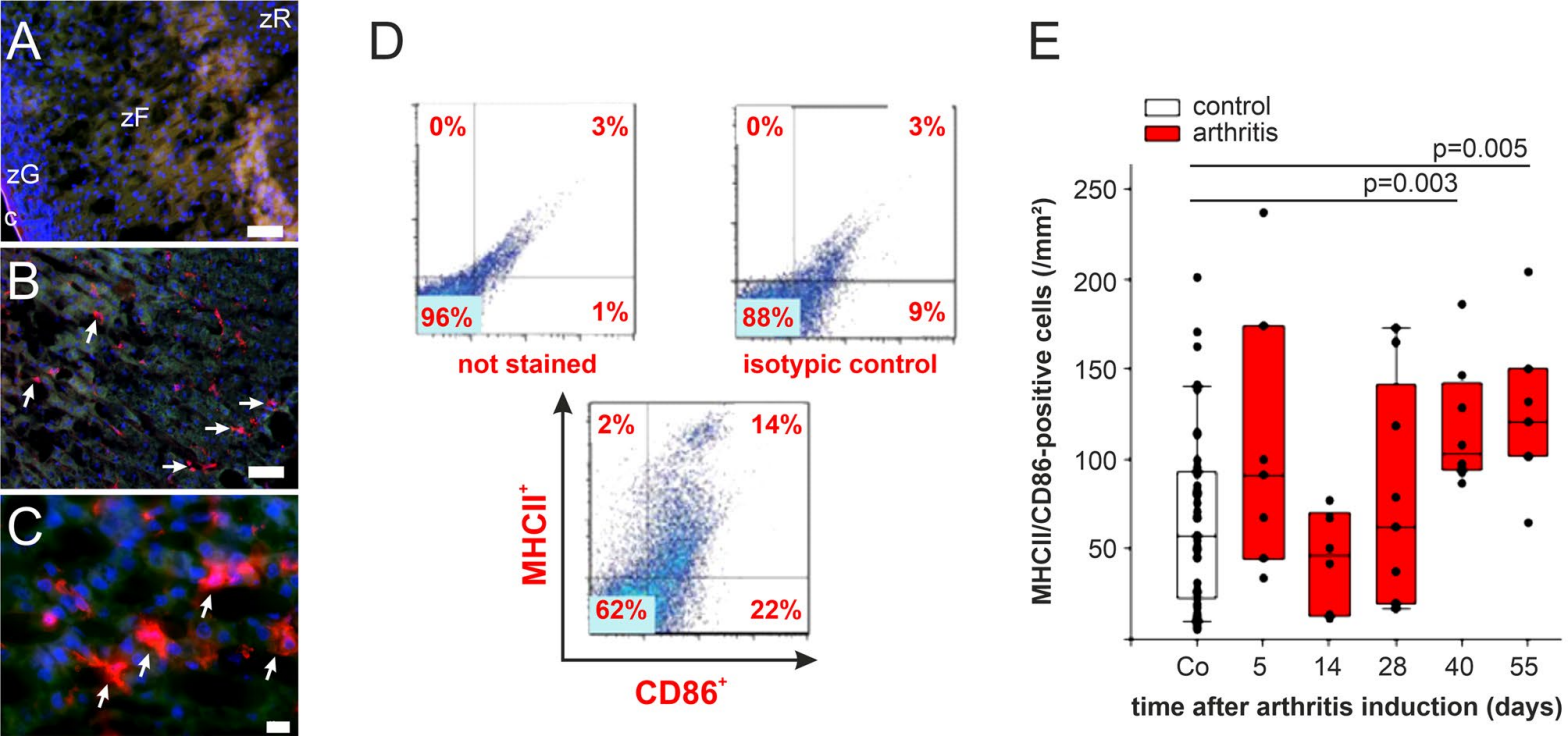
Intraadrenal MHCII+/CD86+ cells during collagen type II induced arthritis (CIA). (**A**) Overview of adrenocortical layers, isotype staining: ×100, bar = 50 µm. (**B**) MHCII+ cells in adrenal cortex (red color, white arrows), ×100, bar = 50 µm. (**C**) MHCII+ adrenocortical cells (red color, white arrows): ×200, bar = 10 µm. (**D**) Quantification of MHCII and CD86 positive adrenal gland cells (exemplary FACS staining) (**E**) Density of intraadrenal MHCII+ cells during the course of CIA. Box plots demonstrate the 10th (whisker), 25th, 50th (median), 75th, and 90th (whisker) percentile. One dot represents the cell density of one rat. *Co* control, *MHCII* major histocompatibility complex II, *c* capsule, *zG* zona glomerulose, *zF* zona fasciculate, *zR* zona reticularis. Red colors in panel (**E**) show results of immunized/arthritic animals.

Above-mentioned results in sJIA prompted us to study the role of IL-1 for adrenal function using the CIA model. Since endogenous tissue-resident macrophages or dendritic cells were good candidates as a cellular source of IL-1 in adrenal glands, immunohistochemistry and FACS analysis were performed on adrenal gland material. It turned out that MHCII+ cells were present in the cortex of the adrenal gland (Fig. 1A–C), while T- and B-cells, and neutrophils were absent (staining identical to isotype staining, which yielded no staining at all). Using separated adrenal cells, presence of MHCII+/CD86+ cells were confirmed by FACS (Fig. 1D). In detailed analyses of the adrenal cortex, these MHCII+ cells were present in healthy controls, and markedly increased during the course of experimental arthritis from day 40 onwards (Fig. 1E; disease starts on day 14 and reaches its maximum on day 28; Ref.^22^).

### MHCII+ cells and bone marrow-derived dendritic cells (BMDCs) decrease corticosterone secretion

**Figure 2:**
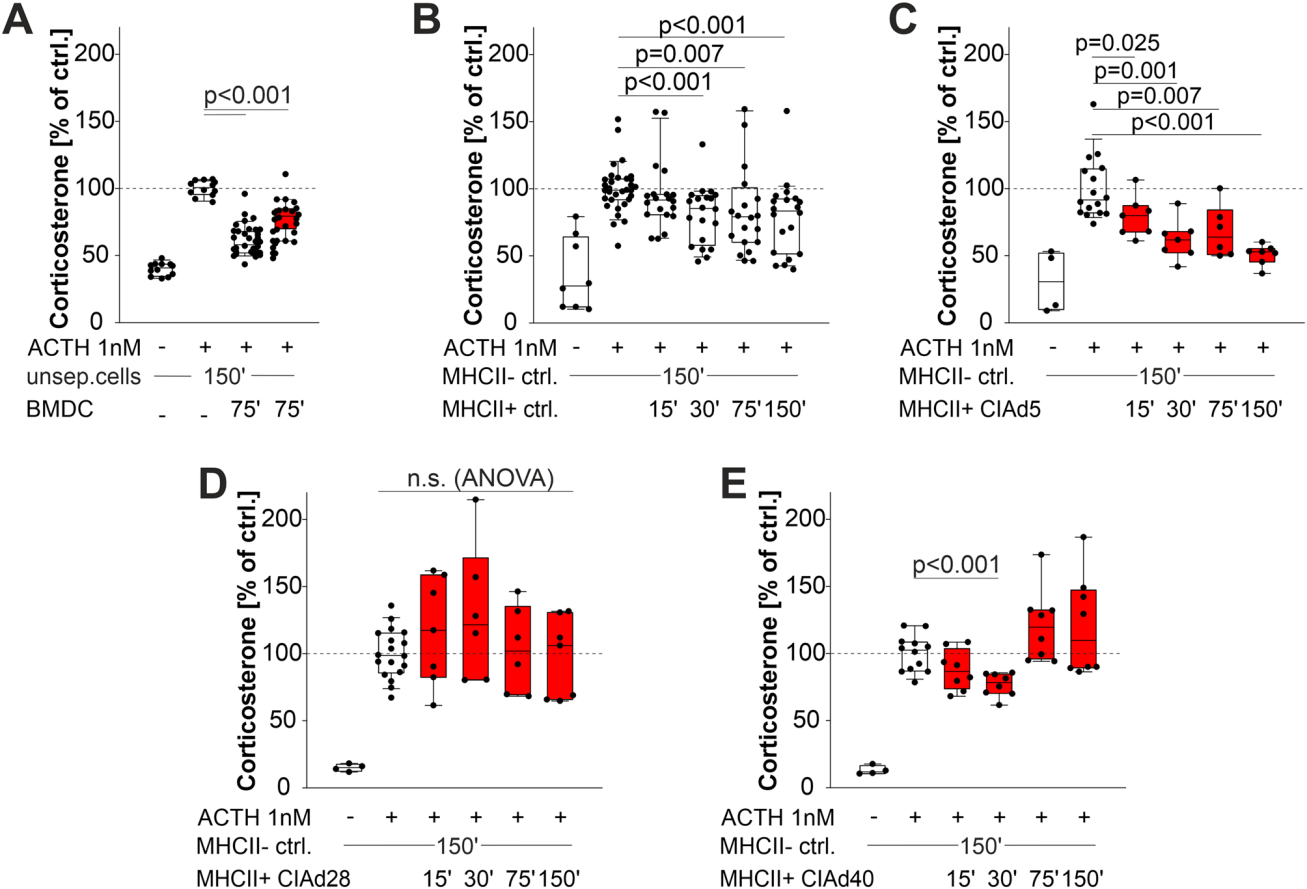
Effects on corticosterone secretion by co-culturing with different cell types. (**A**) Inhibition of corticosterone release from unseparated adrenal gland cells by co-culture with BMDCs obtained from control or arthritic animals (CIA day28). (**B**–**E**) Effect of adding different numbers of intraadrenal MHCII+ cells from control (**B**) or arthritic rats (**C**: day5; **D**: day28; **E**: day40) to 150.000 (150′) MHCII− adrenal gland cells from control animals. Mann–Whitney rank sum test was used to compare two different groups or ANOVA test on ranks to compare all groups. *ACTH* adrenocorticotropic hormone, *BMDCs* bone marrow—derived dendritic cells, *CIA* collagen type II—induced arthritis, *MHCII* major histocompatibility complex II, *unsep*. unseparated, *15′* fifteen thousand (similarly for other numbers of added cells). White (red) boxes show data under control (arthritis) conditions.

In an experimental setup, BMDCs were generated from bone marrow macrophages in order to obtain a pure line of DCs. These BMDCs inhibited ACTH-stimulated corticosterone secretion of unseparated adrenocortical cells (Fig. 2A). In a more detailed analysis, MHCII+ and MHCII− cells were separated from adrenal glands by elaborate magnetic separation techniques.

Co-culture of the two fractions demonstrated that MHCII+ cells inhibited corticosterone secretion of MHCII− cells with an increasing effect depending on MHCII+ cell numbers (Fig. 2B). The inhibiting effect was also observed when cells of arthritic animals of day 5 and day40 were used (Fig. 2C,E). However, on day 28, when maximum arthritis scores were reached (16 of maximum 16 score points, see Ref.^22^), co-culture of MHCII+ and MHCII− cells did not affect corticosterone secretion (Fig. 2D).

In additional experiments, separated MHCII+ cells were given to unseparated adrenal cells that contained endogenous MHCII+ cells. In this setup, the inhibitory effect was not visible under control conditions (Supplementary Fig. 1A), but significantly more so under arthritic conditions (Supplementary Fig. 1B–D).

### IL-1β, IL-18, and the inflammasome play an inhibiting role on corticosterone secretion

**Figure 3:**
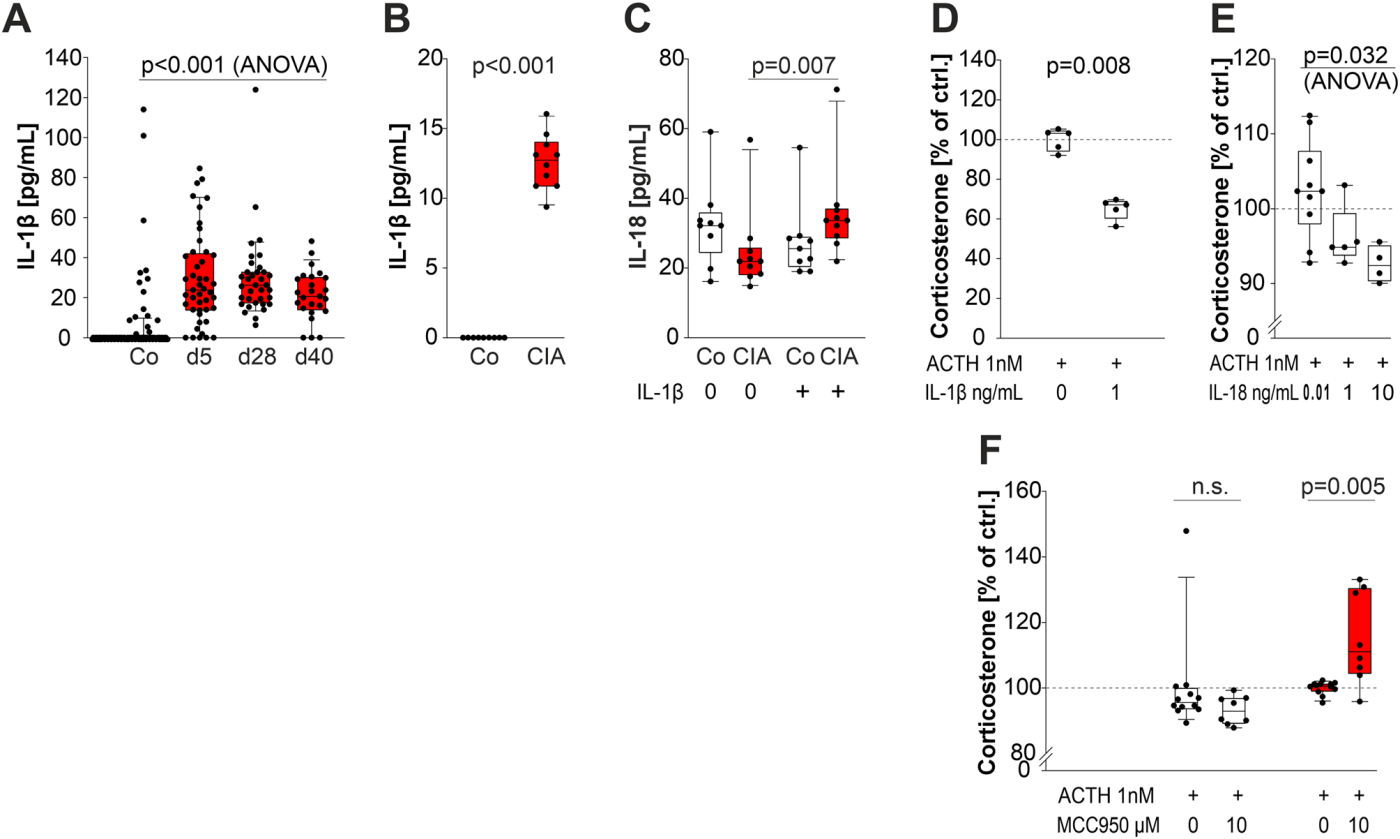
Levels of IL-1β and IL-18 and influence of these cytokines on corticosterone secretion of adrenal gland cells. (**A**) Level of IL-1β in plasma from control and arthritic rats at different time points during arthritis. One dot represents the plasma level of IL-1β in one individual animal. Sample size ranges from n = 25 to 112 per group. (**B**, **C**) Levels of IL-1β (**B**) and IL-18 (**C**) in supernatants of unseparated adrenal gland cells from control (Co, n = 10) and arthritic rats (red box; CIA day28, n = 10). (**D**) and (**E**) Effect of IL-1β (**D**) and IL 18 (**E**) on corticosterone secretion of unseparated adrenal gland cells of control animals. (**F**) Effect of inhibiting the inflammasome with MCC950 (NLRP3-inhibitor) on corticosterone secretion of unseparated adrenal gland cells from either control or arthritic rats (red box; CIA day28). Mann–Whitney rank sum test was used to compare two different groups or ANOVA test on ranks to compare all groups. *ACTH* adrenocorticotropic hormone, *CIA* collagen type II—induced arthritis, *ctrl* control, *IL 1β* interleukin-1β, *IL-18* interleukin-18, *NLRP3* NLR family pyrin domain containing 3.

IL-1β was a good candidate proinflammatory cytokine that might play a role in corticosterone inhibition. In a large sample of rats, plasma levels of IL-1β was clearly elevated during the course of arthritis when compared to control animals (Fig. 3A). Importantly, IL-1β levels stayed high throughout the entire observation period, while plasma-TNF and -IL-10 were only detectable on day 5 after immunization (data not shown).

Using unseparated adrenal gland cells (including MHCII+ and MHCII− cells). IL-1β was much higher in supernatants of cells from arthritic as compared to control animals (Fig. 3B). The difference was not similarly strong for IL-18 (Fig. 3C), but stimulation of adrenal cells with IL-1β increased IL-18 in cell cultures of arthritic animals (Fig. 3C). Thus, IL-1β seems to be a switch that reveals IL-18 effects.

Mimicking an inflammatory condition with IL-1β in cultures with healthy control unseparated adrenal cells, this proinflammatory cytokine markedly inhibited ACTH-stimulated corticosterone secretion (Fig. 3D). Similarly, in these control adrenal cells, IL-18 inhibited ACTH-stimulated corticosterone secretion (Fig. 3E). In supplementary experiments, the NLRP3/inflammasome inhibitor MCC950 increased corticosterone secretion from unseparated adrenal cells of arthritic rats but not of healthy control animals (Fig. 3F).

### The adrenal gland holds a MHCII+ cell-dependent chemokine environment, which increases in arthritis

The presence of MHCII+ cells in the adrenal glands of healthy control and arthritis rats might depend on active migration of these cells into the adrenal glands. This may be more obvious in arthritic compared to control animals (Fig. 1). We hypothesized that chemokines play a decisive role.

Qualitative analyses of chemokine/cytokine presence in supernatants of unseparated adrenal gland cells and BMDCs using proteome profiling revealed top chemokine candidates such as CINC-1,2,3 and LIX (Supplementary Fig. 2), which are chemoattractive for neutrophils^33–35^ and macrophages/dendritic cells^36^. In ELISA studies, presence of these candidates was confirmed, and it turned out that levels were higher in cells of arthritic compared to control animals (Supplementary Fig. 2B,D).

**Figure 4:**
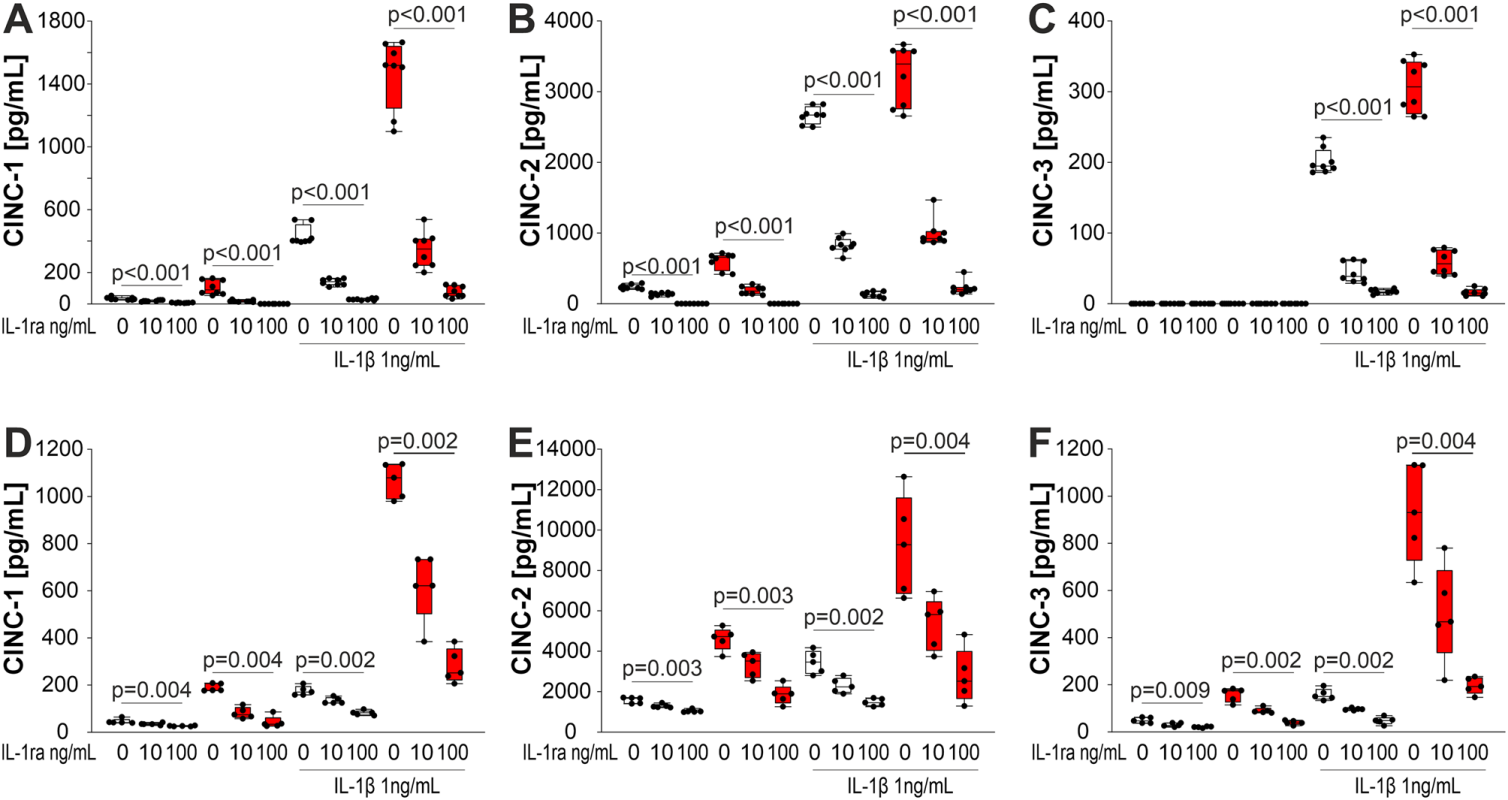
Effect of IL-1β on chemokine profile of unseparated adrenal gland cells and bone marrow derived dendritic cells (BMDCs) of control and arthritic animals (red boxes). (**A**–**C**) Levels of chemokines CINC-1, CINC-2, CINC-3 in supernatants of unseparated adrenal gland cells obtained from arthritic (red boxes) and control animals (white boxes). (**D**–**F**) Levels of chemokines CINC-1, CINC-2, CINC-3 in supernatants of BMDCs obtained from arthritic and control animals. ANOVA on ranks was used to compare groups. *IL-1β* interleukin-1β, *IL-1ra* interleukin 1 receptor antagonist, *CINC* cytokine-induced neutrophil chemoattractant.

Stimulation of these CXC chemokines was largely dependent on IL-1 because this cytokine strongly increased secretion of CINC-1, CINC-2, and CINC-3 from unseparated adrenal gland cells (Fig. 4A–C) and, in a similar way, from BMDCs (Fig. 4D–F). However, even under conditions without additional IL-1 stimulation, IL-1 was important for chemokine secretion because IL-1ra inhibited spontaneous efflux of CXC chemokines from unseparated adrenal gland cells and BMDCs (Fig. 4). This was more marked under arthritic compared to control conditions.

In order to study, whether the above-mentioned CXC chemokines depend on the presence of adrenal MHCII+ cells, MHCII+ cells were co-cultured with the MHCII-negative fraction of adrenal gland cells. Increasing numbers of MHCII+ positive cells led to increasing presence of CXC chemokines CINC-1, CINC-2, CINC-3, and LIX in the supernatants (Supplementary Fig. 3). This indicates that MHCII+ cells are decisive for the presence of these CXC chemokines. In addition, cells from arthritic animals showed increased levels throughout the course of arthritis (Supplementary Fig. 3). Particularly, CINC-2 was highest in arthritic animals of day 28 (Supplementary Fig. 3E versus G).

Importantly, CINC-2 was the only chemokine that slightly increased ACTH-stimulated corticosterone secretion in day 25–28 arthritic animals (Supplementary Fig. 4A). This was not observed for the other chemokines, and the example for LIX is given in Supplementary Fig. 4B.

**Figure 5:**
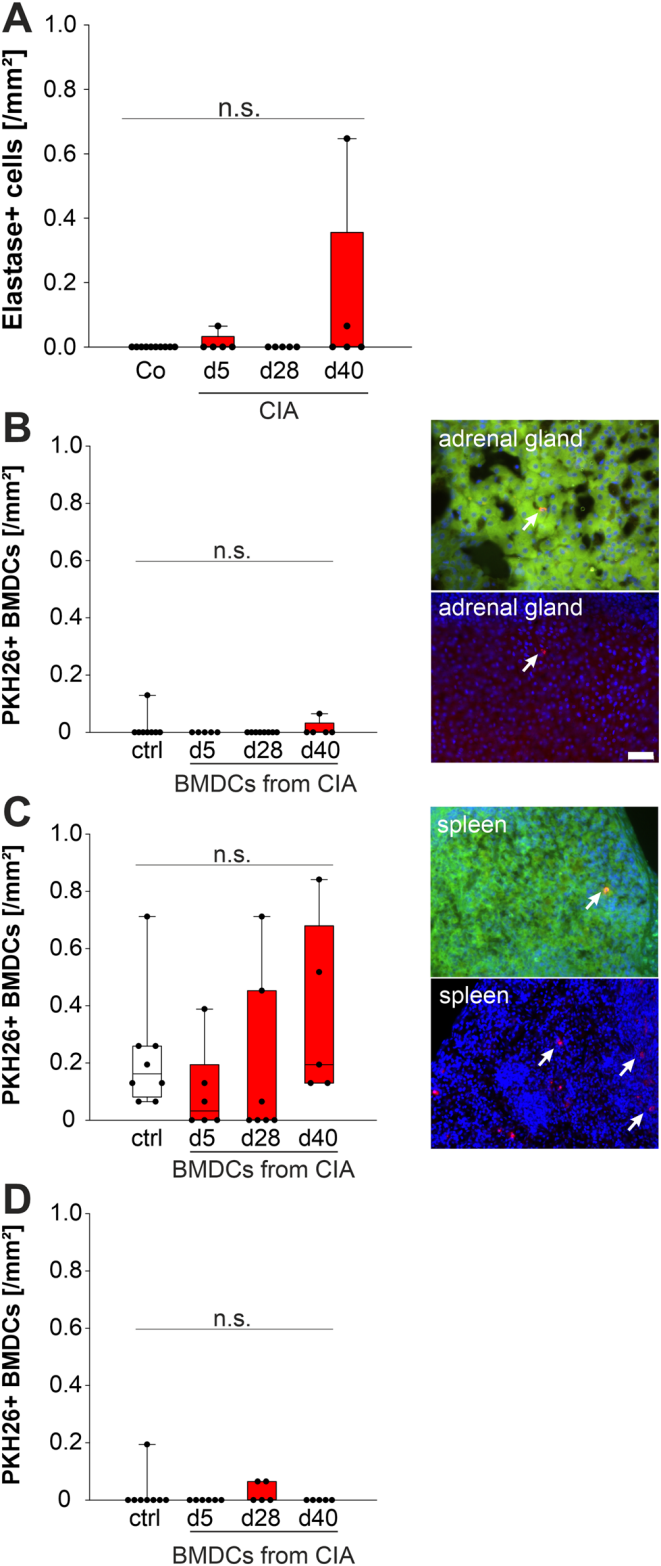
Appearance of leukocytes in adrenal glands, spleen and lymph nodes. (**A**) Spontaneously available neutrophils in adrenal glands of control rats and arthritic rats at different time points during CIA. One dot represents the density of elastase positive cells in the adrenal cortex of one animal. (**B**) Density of injected PKH26+ labeled BMDCs in the adrenal gland (left). Examples of microscope images showing red fluorescent PKH26+ cells (arrows), green fluorescence indicates background, blue indicates DAPI stained cell nuclei. Bar = 50 µm (**C**) Density of injected PKH26+ labeled BMDCs in the spleen (left). Representative microscope images on the right similar like in (**B**). (**D**) Density of injected PKH26+ labeled BMDCs in draining lymph nodes. White (red) boxes show data under control (arthritis) conditions. *BMDC* bone marrow derived dendritic cell, *CIA* collagen type II—induced arthritis, *DAPI* 4′,6-Diamidino-2-phenylindole.

Since MHCII+ cells were present in control and more so in arthritic adrenal glands (Fig. 1), our migration experiments focused on these particular cells. In order to study the appearance of migrating MHCII + DCs, we injected stained BMDCs of control and arthritic animals with different stages of inflammation. Although the number of BMDCs with 10 × 10^6^ cells per animals was high, we did not find many cells in the adrenal gland, spleen and lymph nodes of injected rats, which was independent of control state or arthritis (Fig. 5). These results speak against active arthritis-dependent migration of MHCII+ cells.

### Presence and migration of CXC chemokine-dependent leukocytes into the adrenal gland

Chemokines like CINC-1,2,3, and LIX can be expressed by macrophages^37^ and are important for chemotaxis of neutrophils and monocyte/macrophages^33–36^. However, the number of neutrophils in adrenal gland cortex was very small and independent of control or arthritic conditions (Fig. 5). This was not expected, since others showed that under certain conditions neutrophils are able to migrate into the adrenal glands (see “Discussion”), but our results prevented us from further migration experiments with neutrophils.

## Discussion

This study corroborated the inhibiting role of IL-1 on adrenal gland glucocorticoid secretion in experimental arthritis in rats. It also confirmed increased IL-1 levels in plasma of arthritic animals with otherwise normal corticosterone levels, substantiating the idea of inadequate secretion of this glucocorticoid in relation to IL-1 in humans and rats. This supports the general inhibitory role of IL-1 on endocrine glands, which has so nicely entered clinical thinking in type 2 diabetes mellitus^38–40^.

This present study demonstrated an increase of intraadrenal MHCII-positive cells during the course of collagen type II-induced arthritis. MHCII+ can be present in endocrine glands^16–19^. Thus, these cells might be important in the observed inadequate glucocorticoid secretion. Other groups also demonstrated that MHCII+ cells rapidly appear after induction of highly acute experimental colitis^41^ or after i.v. injection of lipopolysaccharide^17^. These cells play a stimulatory role on adrenal glucocorticoid secretion, but the two models were very acute and short-lived, which cannot be compared to long-standing collagen type II—induced arthritis.

In our model of long-term inflammation MHCII+ cells inhibited corticosterone secretion of adrenal gland cells, and IL-1, IL-18, and the inflammasome are critical for inhibition. While some groups described IL-1 as a corticosteroid stimulatory cytokine, particularly under experimental acute conditions, others observed inhibitory effects of IL-1^13,14,42^. The role of IL-1 is yet not fully defined, and it seems that other framework conditions are important such as the presence of viable MHCII+ cells. Similarly, the expression of intraadrenal IL-18 has been described^43^, yet, its role on glucocorticoid secretion in arthritis is undecided. Moreover, MHCII+ cells produce a myriad of cytokines and membrane molecules that can interfere with corticosterone secretion.

In this study, we found that arthritic compared to control adrenal gland cells produce higher amounts of CXC chemokines, particularly CINC-2, which is strongly dependent on IL-1 and MHCII+ adrenal cells. Thus, CINC-1, CINC-2, CINC-3, and LIX might also be important in chemoattraction of MHCII+ cells, and as in humans, monocytes/macrophages can express the corresponding receptor CXCR2^44^.

Interestingly, MHCII+ cells did not much affect ACTH-stimulated corticosterone secretion in day28 arthritic rats when co-cultured with MHC− adrenal cells (Fig. 2D). This might depend on the fact that CINC-2, which is particularly high in supernatants of CIA adrenal cells on day 28 (Supplementary Fig. 3), has a stimulatory effect on corticosterone secretion (Supplementary Fig. 4). This is the first study to demonstrate a direct effect of a chemokine on adrenal cell corticosterone secretion.

The major role of these chemokines is attraction of neutrophils and MHCII+ cells like monocytes/macrophages/dendritic cells. Importantly, it has been demonstrated that under certain inflammatory settings like addition of IL-1β^45^ or LPS^46^, chemotaxis of neutrophils into the adrenal gland is possible, can be accompanied by chemotaxis of macrophages^47^, and in mice is accompanied by an increase of CXCL2 (the equivalent to rat CINC-3)^48,49^. However, our present study did not find higher levels of neutrophils in adrenal glands of arthritic compared to control animals. Similarly, i.v. injected BMDCs of arthritic animals do not seem to migrate towards the adrenal glands. Technical problems like number of cells or the artificial generation of BMDCs from bone-marrow cells might have obscured directed migration towards the adrenal cortex. It is open to further studies to address this point in more experimental detail.

**Figure 6:**
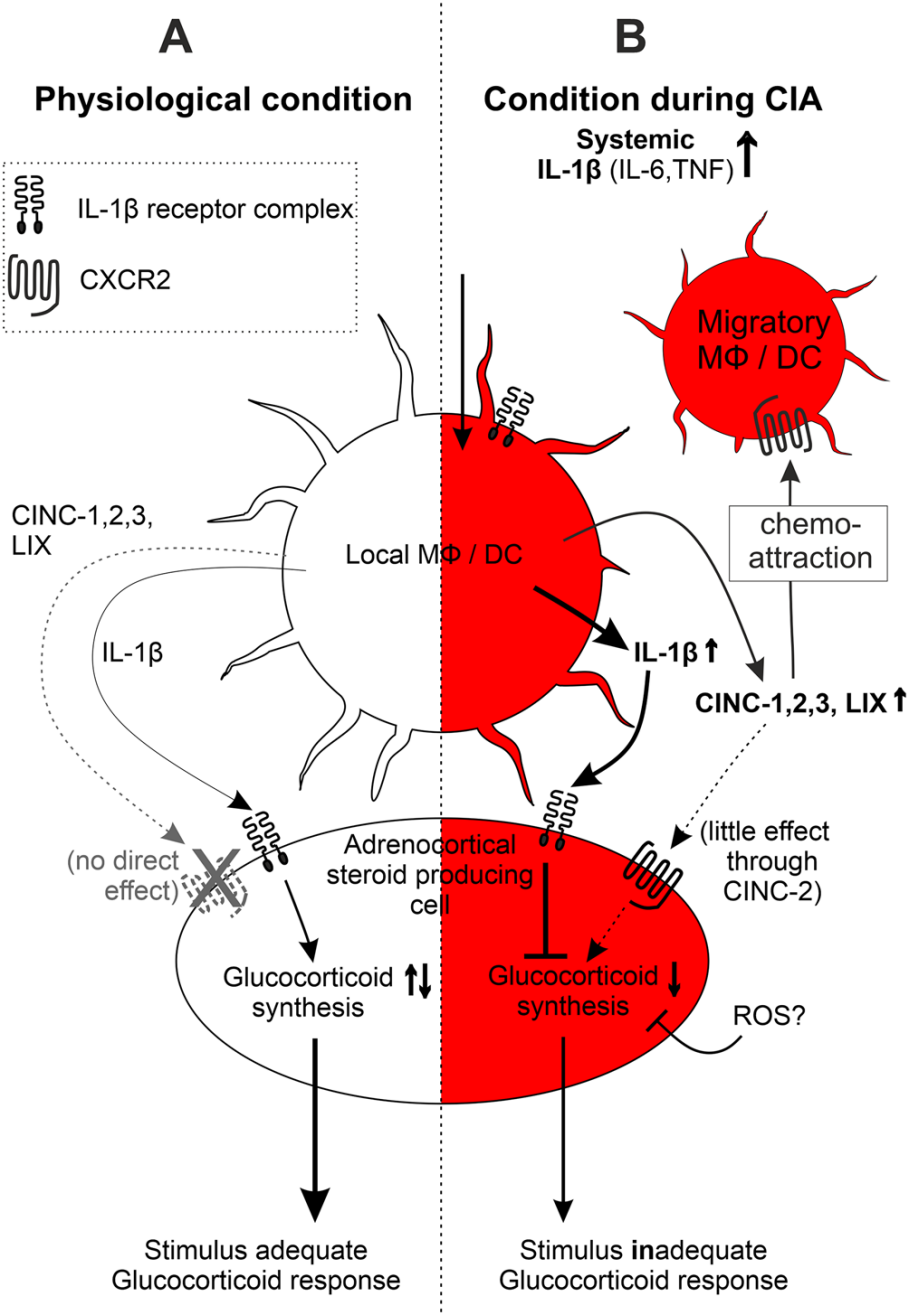
Theoretical model of mechanisms and effects on intraadrenal cells during physiological (**A**, left) and chronic arthritic (**B**, right) conditions. (**A**) In a physiological setting (left), intraadrenal macrophages and dendritic cells via IL-1β do not much influence corticosterone secretion (stimulus adequate glucocorticoid response). (**B**) During chronic inflammatory diseases such as collagen induced arthritis (CIA, right), increased amounts of systemic cytokines such as IL-1β, IL-6 and TNF are able to continuously activate local intraadrenal macrophages, dendritic cells and endothelial cells which in turn release more IL-1β and CXC chemokines CINC-1,2,3 and LIX. Higher amounts of CXC chemokines trigger chemotaxis of migratory cells, which is paralleled by IL 1β—induced inhibition of glucocorticoid secretion (stimulus/inflammation inadequate glucocorticoid response). Higher numbers of activated intraadrenal macrophages and dendritic cells negatively impact glucocorticoid secretion. *MΦ* macrophage, *CXCR2* CXC chemokine receptor 2, *ROS* reactive oxygen species; others see previous figure legends.

In Fig. 6, we demonstrate a possible model that can be relevant for inadequate glucocorticoid secretion during the course of arthritis. In a normal setting, intraadrenal macrophages and dendritic cells via IL-1β do not much influence corticosterone secretion (= stimulus adequate glucocorticoid response). Indeed, IL-1β levels were low in supernatants of control adrenal glands (Fig. 3B). This might largely change during arthritis, and the process might steadily increase. During chronic inflammatory diseases such as collagen induced arthritis (CIA, right), higher amounts of CXC chemokines then trigger chemotaxis of migratory cells, which is paralleled by IL 1β—induced inhibition of glucocorticoid secretion. We called this the stimulus/inflammation inadequate glucocorticoid response. Higher numbers of activated intraadrenal macrophages and dendritic cells would then negatively impact on glucocorticoid secretion (Fig. 6).

The alternative explanation for the observed missing migration of neutrophils and BMDCs might be the following: Increased amounts of systemic cytokines such as IL-1β, IL-6 and TNF possibly activate local intraadrenal macrophages, dendritic cells and endothelial cells. In turn, these cells upregulate markers such as MHCII, CD86, and CD11b, thereby demonstrating an activated state. Increased markers on the surface of local tissue-resident cells, would give the impression of a higher cellular density and a stronger migration towards adrenal glands. However, this second explanation does not explain the role of CXC chemokines that are clearly upregulated during arthritis.

In conclusion, in the rat CIA model, MHCII+ cells have an inhibitory role on corticosterone secretion of adrenal gland cells under control conditions but much more so under in arthritic environment in the adrenal glands. IL-1β is an important inhibitory molecule under these conditions. The role of IL-1 is not yet fully clear and it might well be that this cytokine also has some stimulatory effects under more acute situations or at lower concentrations. In our model of inadequate glucocorticoid secretion during arthritis, IL-1 and chemotactic factors of MHCII+ cells play an important glucocorticoid-inhibiting role.

## Supplementary information

Supplementary Information.

## Data Availability

The corresponding author can submit data and material upon request.

## References

[1] Jessop DS, Harbuz MS (2005). A defect in cortisol production in rheumatoid arthritis: Why are we still looking?. Rheumatology (Oxford).

[2] Straub RH, Bijlsma JW, Masi A, Cutolo M (2013). Role of neuroendocrine and neuroimmune mechanisms in chronic inflammatory rheumatic diseases-The 10-year update. Semin. Arthritis Rheum..

[3] Saldanha C, Tougas G, Grace E (1986). Evidence for anti-inflammatory effect of normal circulating plasma cortisol. Clin. Exp. Rheumatol..

[4] Imrich R (2010). An endocrinologist's view on relative adrenocortical insufficiency in rheumatoid arthritis. Ann. N. Y. Acad. Sci..

[5] Straub RH, Paimela L, Peltomaa R, Schölmerich J, Leirisalo-Repo M (2002). Inadequately low serum levels of steroid hormones in relation to IL-6 and TNF in untreated patients with early rheumatoid arthritis and reactive arthritis. Arthritis Rheum..

[6] Kanik KS (2000). Adrenocorticotropin, glucocorticoid, and androgen secretion in patients with new onset synovitis/rheumatoid arthritis: Relations with indices of inflammation. J. Clin. Endocrinol. Metab..

[7] Mastorakos G, Chrousos GP, Weber JS (1993). Recombinant interleukin-6 activates the hypothalamic-pituitary-adrenal axis in humans. J. Clin. Endocrinol. Metab..

[8] Späth-Schwalbe E (1994). Interleukin-6 stimulates the hypothalamus-pituitary-adrenocortical axis in man. J. Clin. Endocrinol. Metab..

[9] Gisslinger H (1993). Interferon-alpha stimulates the hypothalamic–pituitary–adrenal axis in vivo and in vitro. Neuroendocrinology.

[10] Ehrhart-Bornstein M, Hinson JP, Bornstein SR, Scherbaum WA, Vinson GP (1998). Intraadrenal interactions in the regulation of adrenocortical steroidogenesis. Endocr. Rev..

[11] Jäättelä M, Ilvesmaki V, Voutilainen R, Stenman UH, Saksela E (1991). Tumor necrosis factor as a potent inhibitor of adrenocorticotropin-induced cortisol production and steroidogenic P450 enzyme gene expression in cultured human fetal adrenal cells. Endocrinology.

[12] Straub RH, Härle P, Sarzi-Puttini P, Cutolo M (2006). Tumor necrosis factor-neutralizing therapies improve altered hormone axes: An alternative mode of antiinflammatory action. Arthritis Rheum..

[13] Bornstein SR, Rutkowski H, Vrezas I (2004). Cytokines and steroidogenesis. Mol. Cell Endocrinol..

[14] Herrmann M, Schölmerich J, Straub RH (2002). Influence of cytokines and growth factors on distinct steroidogenic enzymes in vitro: A short tabular data collection. Ann. N. Y. Acad. Sci..

[15] Metzger R, Mempel T, Joppich I, Till H (2000). Organ-specific distribution of major histocompatibility antigens in rats. Pediatr. Surg. Int..

[16] Klein JR (2006). The immune system as a regulator of thyroid hormone activity. Exp. Biol. Med. (Maywood.).

[17] Engstrom L (2008). Systemic immune challenge activates an intrinsically regulated local inflammatory circuit in the adrenal gland. Endocrinology.

[18] Glennon E, Kaunzner UW, Gagnidze K, McEwen BS, Bulloch K (2015). Pituitary dendritic cells communicate immune pathogenic signals. Brain Behav. Immun..

[19] Morris DL (2015). Minireview: Emerging concepts in islet macrophage biology in type 2 diabetes. Mol. Endocrinol..

[20] Renner U, Sapochnik M, Lucia K, Stalla GK, Arzt E (2017). Intrahypophyseal immune-endocrine interactions: Endocrine integration of the inflammatory inputs. Front. Horm. Res..

[21] Straub R, Buttgereit F, Cutolo M (2011). Alterations of the hypothalamic–pituitary–adrenal axis in systemic immune diseases—A role for misguided energy regulation. Clin. Exp. Rheumatol..

[22] Wolff C, Krinner K, Schroeder JA, Straub RH (2015). Inadequate corticosterone levels relative to arthritic inflammation are accompanied by altered mitochondria/cholesterol breakdown in adrenal cortex: A steroid-inhibiting role of IL-1beta in rats. Ann. Rheum. Dis..

[23] Gonzalez-Hernandez JA (1995). IL-1 is expressed in human adrenal gland in vivo. Possible role in a local immune-adrenal axis. Clin. Exp. Immunol..

[24] Judd AM (1998). Cytokine expression in the rat adrenal cortex. Horm. Metab. Res..

[25] del Rey A (2008). Disrupted brain-immune system-joint communication during experimental arthritis. Arthritis Rheum..

[26] Mankowitz L, Rydstrom J, Depierre JW (1991). Adrenocorticotropin-dependent regulation of glutathione transferase subunit 4 in cultured rat adrenal cells. Eur. J. Biochem..

[27] Roskelley C, Auersperg N (1993). Mixed parenchymal-stromal populations of rat adrenocortical cells support the proliferation and differentiation of steroidogenic cells. Differentiation.

[28] Enriquez de Salamanca A, Garcia R (2005). Response of rat fasciculata-reticularis cells in primary culture to bacterial lipopolysaccharide. Microbes Infect..

[29] Grauer O (2002). Analysis of maturation states of rat bone marrow-derived dendritic cells using an improved culture technique. Histochem. Cell Biol..

[30] Muthana M, Fairburn B, Mirza S, Slack LK, Pockley AG (2004). Systematic evaluation of the conditions required for the generation of immature rat bone marrow-derived dendritic cells and their phenotypic and functional characterization. J. Immunol. Methods.

[31] Janelidze S (2005). Activation of purified allogeneic CD4(+) T cells by rat bone marrow-derived dendritic cells induces concurrent secretion of IFN-gamma, IL-4, and IL-10. Immunol. Lett..

[32] Talmor M (1998). Generation or large numbers of immature and mature dendritic cells from rat bone marrow cultures. Eur. J. Immunol..

[33] Shibata F, Konishi K, Nakagawa H (2000). Identification of a common receptor for three types of rat cytokine-induced neutrophil chemoattractants (CINCs). Cytokine.

[34] Takano K, Nakagawa H (2001). Contribution of cytokine-induced neutrophil chemoattractant CINC-2 and CINC-3 to neutrophil recruitment in lipopolysaccharide-induced inflammation in rats. Inflamm. Res..

[35] Madorin WS (2004). Cardiac myocytes activated by septic plasma promote neutrophil transendothelial migration: Role of platelet-activating factor and the chemokines LIX and KC. Circ. Res..

[36] Marsh DR, Flemming JM (2011). Inhibition of CXCR1 and CXCR2 chemokine receptors attenuates acute inflammation, preserves gray matter and diminishes autonomic dysreflexia after spinal cord injury. Spinal Cord.

[37] Shibata F, Shibata Y, Yoshimoto Y, Nakagawa H (2000). The expression of three types of CINCs by lipopolysaccharide-stimulated rat macrophages is inhibited similarly by anti-inflammatory steroids. Inflamm. Res..

[38] Cavelti-Weder C (2016). Development of an interleukin-1beta vaccine in patients with type 2 diabetes. Mol. Ther..

[39] Ehses JA (2009). IL-1 antagonism reduces hyperglycemia and tissue inflammation in the type 2 diabetic GK rat. Proc. Natl. Acad. Sci. U. S. A..

[40] Larsen CM (2007). Interleukin-1-receptor antagonist in type 2 diabetes mellitus. N. Engl. J. Med..

[41] Franchimont D (2000). Adrenal cortical activation in murine colitis. Gastroenterology.

[42] Tkachenko IV, Jaaskelainen T, Jaaskelainen J, Palvimo JJ, Voutilainen R (2011). Interleukins 1alpha and 1beta as regulators of steroidogenesis in human NCI-H295R adrenocortical cells. Steroids.

[43] Sugama S, Conti B (2008). Interleukin-18 and stress. Brain Res. Rev..

[44] Bonecchi R (2000). Induction of functional IL-8 receptors by IL-4 and IL-13 in human monocytes. J. Immunol..

[45] Buss NA, Gavins FN, Cover PO, Terron A, Buckingham JC (2015). Targeting the annexin 1-formyl peptide receptor 2/ALX pathway affords protection against bacterial LPS-induced pathologic changes in the murine adrenal cortex. FASEB J..

[46] Butler LD (1989). Interleukin 1-induced pathophysiology: Induction of cytokines, development of histopathologic changes, and immunopharmacologic intervention. Clin. Immunol. Immunopathol..

[47] Amanzada A (2014). Induction of chemokines and cytokines before neutrophils and macrophage recruitment in different regions of rat liver after TAA administration. Lab. Investig..

[48] Kanczkowski W (2013). Characterization of the LPS-induced inflammation of the adrenal gland in mice. Mol. Cell Endocrinol..

[49] Kanczkowski W (2013). Hypothalamo-pituitary and immune-dependent adrenal regulation during systemic inflammation. Proc. Natl. Acad. Sci. U. S. A..

