# Supplementary material for: MHC/class-II-positive cells inhibit corticosterone of adrenal gland cells in experimental arthritis: a role for IL-1β, IL-18, and the inflammasome
Source: Sci Rep. 2020 Oct 13;10:17071. doi: 10.1038/s41598-020-74309-0 (PMC7554037; doi:10.1038/s41598-020-74309-0)
Supplement: Supplementary file 1 — Supplementary Information. [file 41598_2020_74309_MOESM1_ESM.pdf]

Supplementary material

Running Head: Adrenal dendritic cells/macrophages block glucocorticoid secretion in arthritis

MHC/class-II-positive cells inhibit corticosterone of adrenal gland cells in experimental arthritis - a role for IL-1β, IL-18, and the inflammasome

Hubert Stangl, Anita Krammetsvogl, Martin Lesiak, Christine Wolff, Rainer H. Straub*

Laboratory of Experimental Rheumatology and Neuroendocrine Immunology, Department of Internal Medicine, University Hospital, Regensburg, Germany

***Corresponding author**
Rainer H. Straub
Laboratory of Experimental Rheumatology and Neuroendocrine Immunology
Department of Internal Medicine
University Hospital Regensburg
Biopark I, Am Biopark 9
93053 Regensburg, Germany
Phone: +49 941 944 7120
Email: rainer.straub@ukr.de

Total number of words: 2991

Emails
hubert.stangl@ukr.de
anita.krammetsvogl@gmx.de
martin.lesiak@uk-augsburg.de
christine.wolff@barmherzige-regensburg.de
rainer.straub@ukr.de

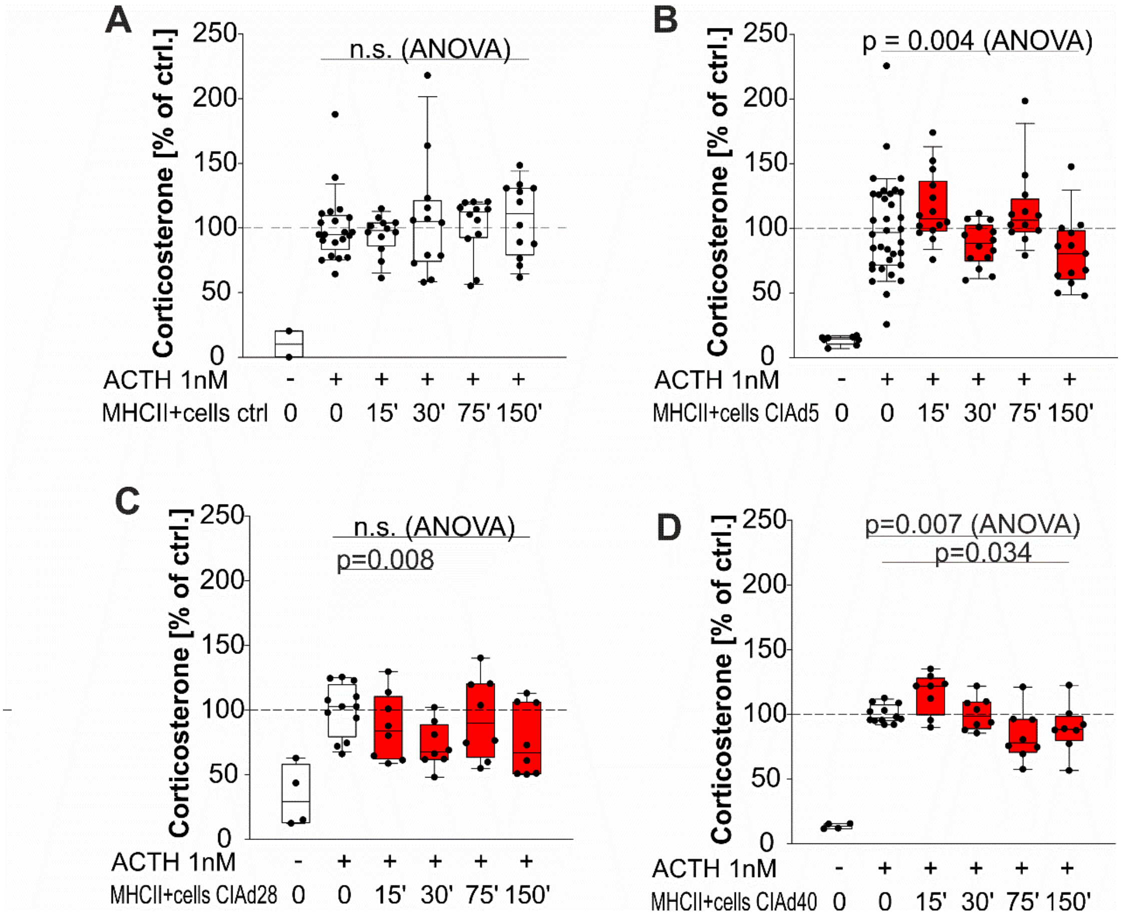

Suppl. Fig. 1

Effects on corticosterone secretion by co-culturing unseparated adrenal gland cells from healthy control animals with MHCII+ cells derived from control (A) or arthritic adrenal glands (B-D). A) Effect on corticosterone release from 150.000 unseparated adrenal gland cells by co-culture with healthy control intraadrenal MHCII+ cells. B-D) Effect on corticosterone release from 150.000 unseparated adrenal gland cells by co-culture with intraadrenal MHCII+ cells obtained from arthritic rats at different time points in CIA (B: CIA day5; C: CIA day28; D: CIA day40). ANOVA on ranks test was used to compare many groups, rank sum test was used to compare two groups. White (red) boxes show data under control (arthritis) conditions.Abbreviations: see previous figure legends in main part.

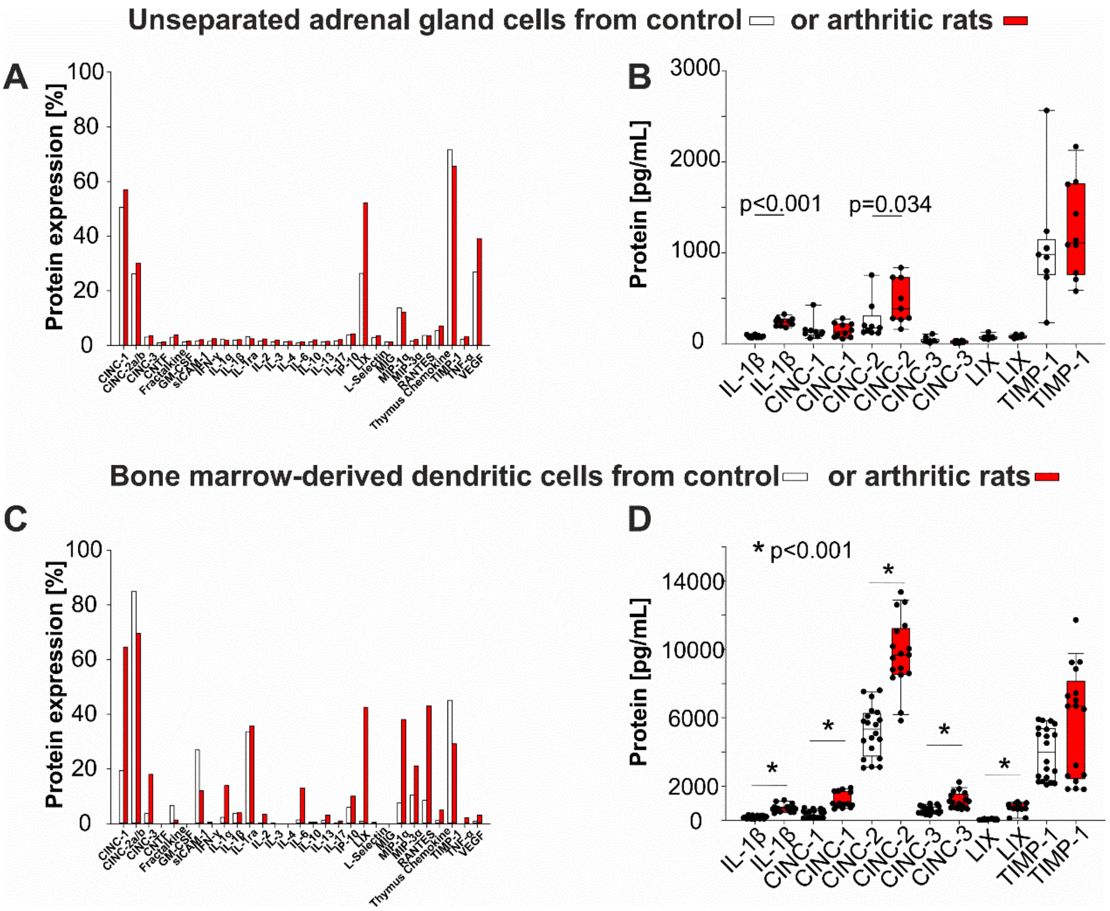

Suppl. Fig. 2 Cytokine profile of unseparated adrenal gland cells (A,B) and BMDCs (C,D) from control and arthritic animals. A) Relative cytokine expression (proteome profiler) in supernatants from unseparated adrenal gland cells from control (white color) and arthritic (CIA day25) rats (red color). B) Cytokine concentrations (ELISA) in supernatants from unseparated adrenal gland cells from control and arthritic (red color, CIA day25) rats. C) Relative cytokine expression (proteome profiler) of supernatants from differentiated BMDCs from control (white color) and arthritic (red color, CIA day25) rats. D) Cytokine concentrations (ELISA) in supernatants from BMDCs from control (white color) and arthritic (red color, CIA day25-28) rats.

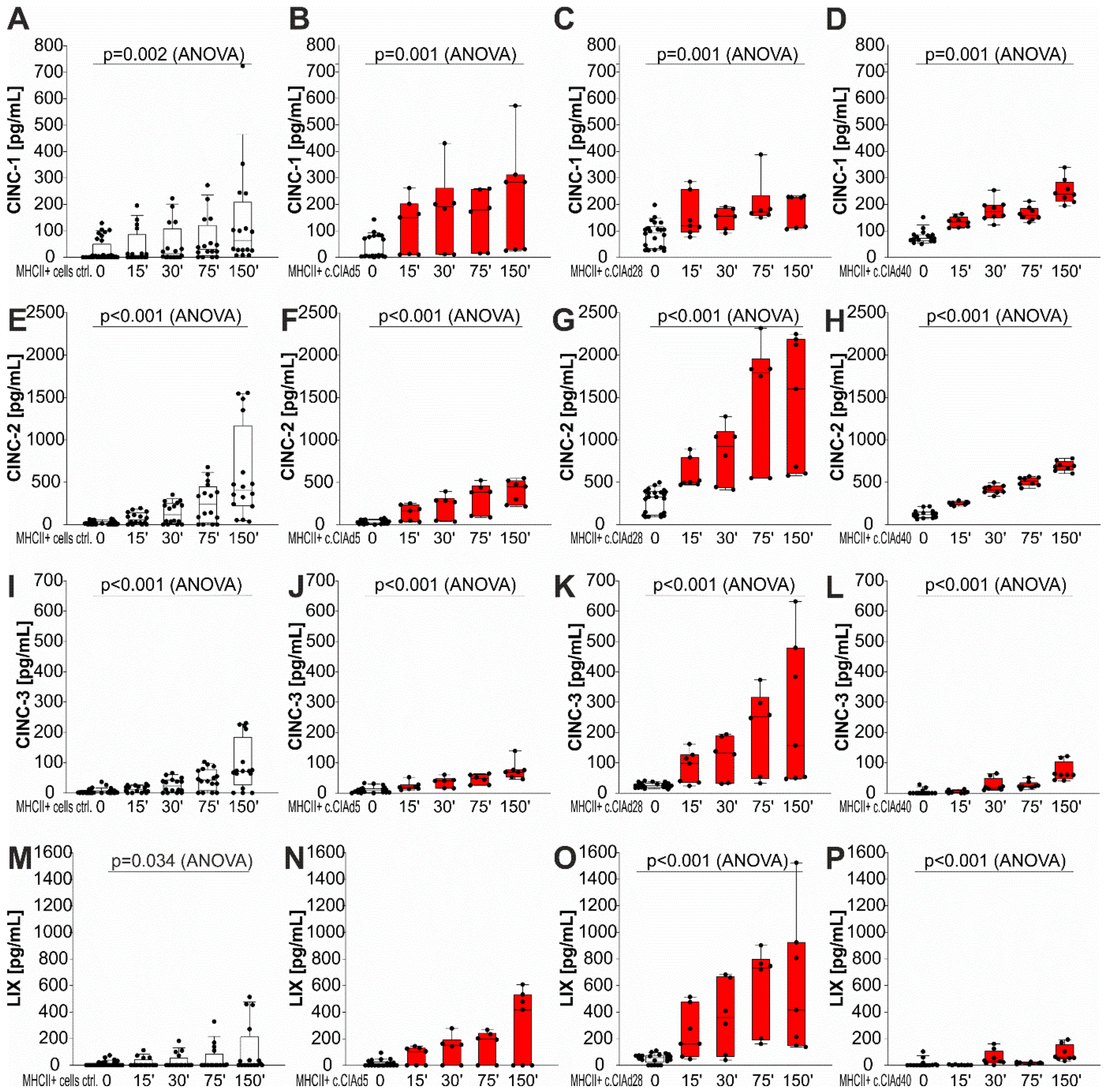

Suppl. Fig. 3 Cytokine profile in supernatants of co-culture experiments with MHCII negative (-) adrenal gland cells of control rats together with MHCII positive (+) adrenal gland cells from control animals (white color) or rats with CIA (red color) at different time points. Under all conditions, 150.000 MHCII negative (-) adrenal gland cells were used. MHCII+ cells were added according to the given numbers (e.g., 15', 15.000). A D) Levels of CINC-1 in experiments with MHCII+ cells obtained from control (A), CIA day5 (B), CIA day28 (C), or CIA day40 (D). The same supernatants were assayed for levels of CINC-2 (E-H), CINC-3 (I-L), and LIX (M-P), respectively. ANOVA on ranks test was used to compare all groups. Abbreviations: see previous figure legends.

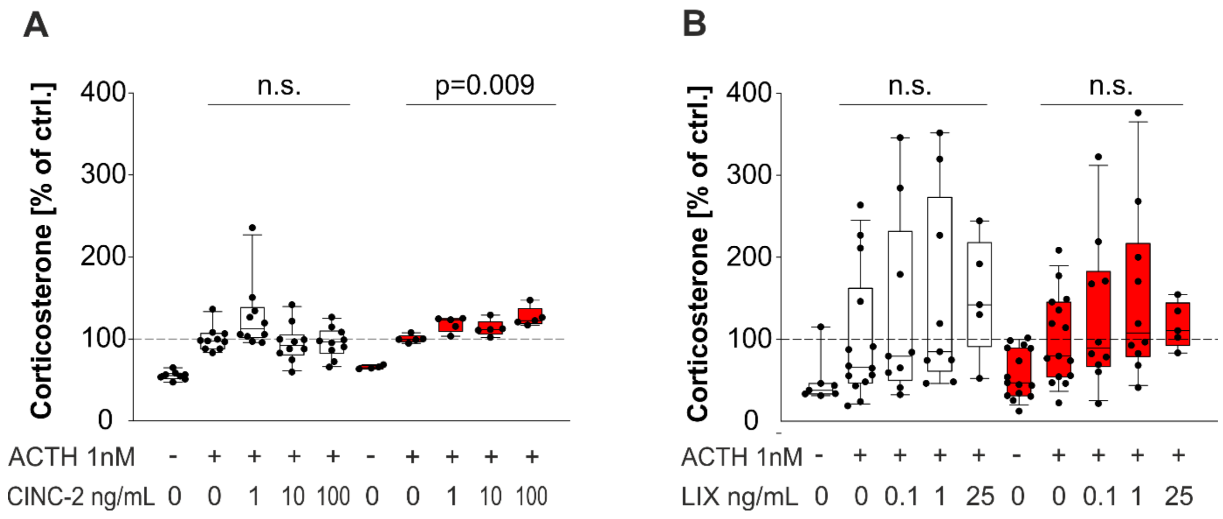

Suppl. Fig. 4 Effect of CXC chemokines on corticosterone secretion of unseparated adrenal gland cells from control (white color) or arthritic rats (red color). A) Effect of increasing amounts of CINC-2 on corticosterone secretion from unseparated adrenal gland cells from controls and arthritic rats (CIA day 25-28). B) Effect of increasing amounts of LIX on corticosterone secretion from unseparated adrenal gland cells obtained from controls and arthritic rats (CIA day 25-28). ANOVA on ranks test was used to compare all groups. Abbreviations: n.s., not significant; others see previous figure legends in main part.
